# Recommendations for optimal interdisciplinary management and healthcare settings for patients with rare neurological diseases

**DOI:** 10.1186/s13023-024-03023-1

**Published:** 2024-02-13

**Authors:** Holm Graessner, Carola Reinhard, Tobias Bäumer, Annette Baumgärtner, Knut Brockmann, Norbert Brüggemann, Eva Bültmann, Jeanette Erdmann, Kirstin Heise, Günter Höglinger, Irina Hüning, Frank J. Kaiser, Christine Klein, Thomas Klopstock, Ingeborg Krägeloh-Mann, Markus Kraemer, Kerstin Luedtke, Martin Mücke, Thomas Musacchio, Andreas Nadke, Alma Osmanovic, Gabriele Ritter, Katharina Röse, Christopher Schippers, Ludger Schöls, Rebecca Schüle, Jörg B. Schulz, Joachim Sproß, Eveline Stasch, Gilbert Wunderlich, Alexander Münchau

**Affiliations:** 1 Institute for Medical Genetics and Applied Genomics, University of Tübingen, Tübingen, Germany; 2 Centre for Rare Diseases, University Hospital Tübingen, Tübingen, Germany; 3 Institute of Systems Motor Science, University of Lübeck, Lübeck, Germany; 4 Center for Rare Diseases, University of Lübeck and University Hospital Medical Center, Schleswig-Holstein, Germany; 5 Institute of Health Sciences, University of Lübeck, Lübeck, Germany; 6 Interdisciplinary Pediatric Center for Children With Developmental Disabilities and Severe Chronic Disorders, Department of Pediatrics and Adolescent Medicine, University Medical Center, Göttingen, Germany; 7 Department of Neurology, University Hospital Medical Center Schleswig-Holstein, Schleswig-Holstein, Germany; 8 Institute of Neurogenetics, University of Lübeck, Lübeck, Germany; 9 Institute of Diagnostic Und Interventional Neuroradiology, Hannover Medical School, Carl-Neuberg-Str. 1, 30625 Hannover, Germany; 10 Institute for Cardiogenetics, DZHK (German Research Centre for Cardiovascular Research), Partner Site Hamburg/Lübeck/Kiel, and University Heart Center Lübeck, University of Lübeck, Lübeck, Germany; 11 Department of Health Sciences and Research, Medical University of South Carolina, Charleston, USA; 12 Department of Movement Sciences, Movement Control and Neural Plasticity Research Group, KU Leuven, Louvain, Belgium; 13 Department of Neurology, Hannover Medical School, Carl-Neuberg-Straße 1, 30625 Hannover, Germany; 14 Department of Neurology, Ludwig-Maximilians-Universität München, Marchioninistraße 15, 81377 Munich, Germany; 15 German Center for Neurodegenerative Diseases (DZNE), Feodor-Lynen-Straße 17, 81377 Munich, Germany; 16 Institute of Human Genetics, University of Lübeck, Lübeck, Germany; 17 Essen Center for Rare Diseases (Essener Zentrum Für Seltene Erkrankungen, EZSE), University Hospital Essen, Essen, Germany; 18 Institute of Neurogenetics, University of Lübeck and University Hospital Medical Center , Schleswig-Holstein, Germany; 19 Friedrich Baur Institute at the Department of Neurology, University Hospital, LMU , Munich, Germany; 20 Munich Cluster for Systems Neurology (SyNergy), Munich, Germany; 21 German Center for Neurodegenerative Diseases (DZNE), Munich, Germany; 22 Department of Pediatric and Developmental Neurology, Social Pediatrics, University of Tübingen, Tübingen, Germany; 23 Department of Neurology, Alfried Krupp Hospital Essen, Essen, Germany; 24 Department of Neurology, Medical Faculty, Heinrich Heine University, Düsseldorf, Germany; 25 Department of Physiotherapy, Institute of Health Sciences, University of Lübeck, Lübeck, Germany; 26 Institute for Digitalization and General Medicine, RWTH Aachen University, Pauwelsstraße 30, 52074 Aachen, Germany; 27 Center for Rare Diseases Aachen (ZSEA), RWTH Aachen University, Pauwelsstraße 30, 52074 Aachen, Germany; 28 Department of Neurology, RWTH Aachen University, Pauwelsstraße 30, 52074 Aachen, Germany; 29 Department of Neurology, University Hospital of Würzburg, Josef-Schneider-Str. 11, 97080 Würzburg, Germany; 30 Center for Rare Diseases, University Hospital of Würzburg, Josef-Schneider-Str. 2, 97080 Würzburg, Germany; 31 Deutsche Heredo-Ataxie-Gesellschaft E.V., Stuttgart, Germany; 32 Klinikum Ameos, 23774 Heiligenhafen Friedrich Ebertstrrasse 100 Huntington Zentrum Nord, Heiligenhafen, Germany; 33 Center for Rare Diseases Aachen (ZSEA), University Hospital Aachen, Aachen, Germany; 34 Department of Neurology and Hertie-Institute for Clinical Brain Research, University of Tübingen, Tübingen, Germany; 35 German Center of Neurodegenerative Diseases (DZNE), Tübingen, Germany; 36 Division of Neurodegenerative Diseases, Department of Neurology, Heidelberg University Hospital and Faculty of Medicine, Heidelberg, Germany; 37 Center for Neurology and Hertie Institute for Clinical Brain Research, University of Tübingen, Tübingen, Germany; 38 German Center for Neurodegenerative Diseases (DZNE), Tübingen, Germany; 39 Department of Neurology and Center for Rare Diseases, University Hospital, RWTH Aachen University, Aachen, Germany; 40 Deutsche Gesellschaft Für Muskelkranke e.V. Im Moos 4, 79112 Freiburg, Germany; 41 Deutsche PSP-Gesellschaft e.V. Weingartenstr. 28a, 61231 Bad Nauheim, Germany; 42 Faculty of Medicine and University Hospital, Department of Neurology, University of Cologne, Cologne, Germany

**Keywords:** Rare neurological diseases, Optimal care, Interdisciplinary management, Healthcare settings

## Abstract

**Background:**

In 2017, the German Academy for Rare Neurological Diseases (Deutsche Akademie für Seltene Neurologische Erkrankungen; DASNE) was founded to pave the way for an optimized personalized management of patients with rare neurological diseases (RND) in all age groups. Since then a dynamic national network for rare neurological disorders has been established comprising renowned experts in neurology, pediatric neurology, (neuro-) genetics and neuroradiology. DASNE has successfully implemented case presentations and multidisciplinary discussions both at yearly symposia and monthly virtual case conferences, as well as further educational activities covering a broad spectrum of interdisciplinary expertise associated with RND. Here, we present recommendation statements for optimized personalized management of patients with RND, which have been developed and reviewed in a structured Delphi process by a group of experts.

**Methods:**

An interdisciplinary group of 37 RND experts comprising DASNE experts, patient representatives, as well as healthcare professionals and managers was involved in the Delphi process. First, an online collection was performed of topics considered relevant for optimal patient care by the expert group. Second, a two-step Delphi process was carried out to rank the importance of the selected topics. Small interdisciplinary working groups then drafted recommendations. In two consensus meetings and one online review round these recommendations were finally consented.

**Results:**

38 statements were consented and grouped into 11 topics: health care structure, core neurological expertise and core mission, interdisciplinary team composition, diagnostics, continuous care and therapy development, case conferences, exchange / cooperation between Centers for Rare Diseases and other healthcare partners, patient advocacy group, databases, translation and health policy.

**Conclusions:**

This German interdisciplinary Delphi expert panel developed consented recommendations for optimal care of patients with RND in a structured Delphi process. These represent a basis for further developments and adjustments in the health care system to improve care for patients with RND and their families.

**Supplementary Information:**

The online version contains supplementary material available at 10.1186/s13023-024-03023-1.

## Introduction

In Europe, a disease is considered “rare” when affecting < 1 person in 2000. Although rare diseases (RDs) have—per definition—a low prevalence, the total number of patients with a RD is high, affecting about 3.5–5.9% of the population equating to 263–446 million people affected globally at any point in time [1]. The majority of RD have neurological manifestations including the central and peripheral nervous system and muscles [2]. In Germany alone, we estimate the number of patients with rare neurological diseases (RND) to amount to approximately 150,000 cases with 7000–8000 new cases manifesting each year. Care of patients with RND concerns a considerable fraction of the healthcare service provided by a national healthcare system [3]. Thus, suboptimal management of RND patients causes major healthcare problems [4].

The German Academy for Rare Neurological Diseases (Deutsche Akademie für Seltene Neurologische Erkrankungen; DASNE) is a German initiative aiming at paving the way for an optimized personalized management of patients with RND in all age groups. Instigated by the Centers for Rare Diseases in Lübeck and Tübingen, a dynamic national network for RND has been constituted comprising renowned experts in the fields of neurology, pediatric neurology, pediatrics, (neuro-)genetics and neuroradiology. DASNE has successfully implemented case presentations and multidisciplinary discussions both at yearly symposia and monthly virtual case conferences, as well as further educational activities covering a broad spectrum of interdisciplinary expertise associated with RND. [5]. The DASNE is associated with the German Reference Network (Deutsches Referenznetzwerk; DRN) for Rare Neurological Diseases, founded in 2021.

Taking into account both the magnitude of the healthcare challenge to provide optimal care for RND patients as well as the ambition of the DASNE and the German Reference Network for RND, precisely determining what optimal interdisciplinary management and healthcare settings for patients with rare neurological diseases mean is warranted. In the present study, following a structured Delphi process, we developed and reviewed 38 recommendation statements by an interdisciplinary expert group composed of patient representatives, DASNE experts, as well as other healthcare professionals and managers. The development of the recommendations has been undertaken by thematic groups of experts (e.g. continuous care and therapy development, health policy) followed by a consensus meeting with the whole group of experts. Colleagues with different fields of expertise and backgrounds were involved. The recommendations were finalized in November 2021 and endorsed by the entire interdisciplinary expert group. Some are specific for RND, some are generically applicable also to other rare diseases. Furthermore, the specific areas, for which statements were developed, have a direct connection to care services or cover care related overarching topics such as health policy.

The aim of the recommendation is to refer to care for RND patients in general. Hence, specific recommendations for particular diseases or disease groups such as ataxias or leukodystrophies, are not covered in these statement recommendations.

The recommendations are conceived as action statements for the management and provision of clinical care, they are not merely political or contemplative. They refer to the structure of the care facility, ensuring neurological core expertise and core mission, composition of the interdisciplinary team, diagnostics, case conferences, continuous care and therapy development, translation, patient advocate groups, health policy, exchange/cooperation between rare disease centers and other partners in the health sector, and to databases. Research of RND is not addressed specifically. However, for RD the boundaries of what relates to care and what to research are often hard to define.

## Results

37 experts, chosen for their involvement in DASNE, expertise in RND and representing the interdisciplinary team involved in RND care, collaborated to develop this recommendation. Involved expertise included neurology, pediatric neurology, human genetics, neuroradiology, neurorehabilitation, social counseling and patient advocacy groups as well as two directors of German rare disease centers. A Delphi-like consensus methodology was adopted. A systematic PubMed search yielded no results as to similar studies specifically addressing the topic of this study.

### Topics of relevance for optimal care of RND patients

An online collection of topics considered relevant for optimal patient care of RND patients was performed by the expert group. Next, a two-step Delphi process was performed to rank the importance of the selected topics. The two Delphi rounds revealed that none of the topics were rated as not important, the lowest median voting received on a Likert scale from 1 (not important) to 6 (most important) was 4. Table 1 shows the main topics as well as those subtopics that reached a median score ≥ 5 and were, thus, included in the further development of the statements. The full ranking results are given as Appendix 1. The selected eleven main topics reflect the full spectrum of topics that influence the quality of care provided to RND patients and include infrastructural, care as well as policy topics.

**Table 1:** Main topics and subtopics with a median ≥ 5 in the second Delphi round

Structure of the care facility	
Remuneration/available time	
Spatial equipment incl. therapy rooms	
Integration into the health care system/establishment of cross-sector care pathways	

Ensuring neurological core expertise and core mission	
Specialized training	
Continued education and training	
Promotion of young talent	
Expertise for specific rare diseases/disease groups	

Composition of the interdisciplinary team	
Neurology, Neuropediatrics, Cognitive Neurology, Neurogenetics, Neuropathology	
Neuroradiology/Nuclear Medicine	
Speech therapy, occupational therapy, physiotherapy	
Nursing care	
(Neuro)Psychology	
Psychosocial/social-medical counseling	
Genetic counseling	

Diagnostics	
Next Generation Sequencing including reimbursement and evaluation	

Case conferences	
On site case conferences	

Continuous care and therapy development	
Interdisciplinary planning	
Clinical trials	
Standardized scales and scores	
Quality of life	

Translation	
Patient advocacy organizations	
Health policy	
Social discourse on diagnostics and treatment costs	
Political lobbying	
Gene Therapy	

Exchange and cooperation between rare disease centers and other partners in the health care sector	
Exchange and cooperation between expertise centers for rare diseases	
Cross-sectoral exchange and cooperation	

Databases	
Collaborative registries of rare disease centers	
Registries focused on specific disease	

### Consensus recommendations for optimal interdisciplinary management and healthcare settings for patients with RND

Small interdisciplinary working groups comprising three to five experts were formed and tasked to draft recommendations for the eleven main topics taking particularly considering the selected subtopics. In two subsequent consensus meetings and one online review round these recommendations were finally consented. Table 2 shows the main points of the consensus recommendations for optimal interdisciplinary management and healthcare settings for patients with RND.

**Table 2:** Key points of consensus recommendations for optimal interdisciplinary management and healthcare settings for patients with RND

Heading of consensus statements	Key points of the corresponding consensus recommendation
Structure of the care facility (Statements 1–3)	Specialist out-patient clinics Interdisciplinary and interprofessional care, i.e. joint clinics of neurology / pediatrics / neurosurgery / orthopedics / rehabilitation / occupational therapy and physiotherapy NAMSE and ERN-RND standards should be fulfilled Curriculum for RND including rotations to specialist out-patient clinics
Ensuring neurological core expertise and core mission (Statements 4–6)	Neurologic and neurogenetic expertise in RND Engagement of medical students / residents
Composition of the interdisciplinary team (Statements 7–11)	Interdisciplinary and interprofessional team including medicine (neurology, pediatric neurology, neurogenetics, neuropathology, neuroradiology), health care professions (speech therapy, occupational therapy, physiotherapy, (neuro)psychology), specialist nurses, psychosocial/social medical counseling CME and interprofessional education Patient centered approach with involvement of patients' relatives and representatives, and patient organizations; shared decision making Focus on goal-attainment and empowerment Networking with other de-centralized outpatient care (e.g. general practitioners, outpatient physiotherapy practices) including case conferences Availability of digital infrastructure
Diagnostics (Statements 12–13)	Interdisciplinary and interprofessional internal / regional / national / international case conferences that should be planned and documented according to SOPs Exome sequencing considered genetic analysis of choice; indication should be based on interdisciplinary case conferences with participation of human genetics Neuroradiology with expertise in the field of RND Clear and structured communication of results of diagnostic procedures Diagnostic endeavors targeted to possible therapeutic consequences
Case conferences (Statements 14–16)	Interdisciplinary and interprofessional case conferences according to SOPs SOPs should be harmonized across centers in the DRN for RND in collaboration with the DASNE Minimum requirements: three different specialties, mandatory participation of neurology Structured case presentations, documentation in the local hospital information / management system Format can be on-site or as video conference that should be easy-to-use and follow European data protection standards Remuneration according to number of disciplines involved External experts should receive personal compensation
Continuous care and therapy development (Statements 17–22)	Structured and validated information on centers in the Internet Early consultation of RND experts by practicing neurologists / general practitioners through remunerated participation in (online) case conferences that are credited withCME points Reducing budget restrictions for general practitioners for patients with rare diseases Interdisciplinary and interprofessional care networks for specific RND National registries / cohort studies to create trial-ready cohorts National platforms for communication and standardization of individual healing attempts
Translation (Statements 23–24)	Early diagnosis including newborn screening prerequisite for the development of targeted treatment Identification of biochemical biomarkers and neuroimaging parameters Development of recommendations for clinical description and clinically meaningful and appropriate outcome parameters
Patient advocacy organizations (Statements 25–27)	Shared informed decisions of treating physicians, affected patients and their families or carers Continuous care by experienced doctors with regular specialized consultations Emergency treatment and inpatient admissions in specialized centers
Health policy (Statements 28–30)	Expertise based decisions; communication of expertise to political and administrative decision makers with the involvement of patient organizations Across sector-care including cross-sector conferences and patient files with adequate remuneration for participants Interdisciplinary interprofessional care both for children / adolescents and adults. In adults, centers akin to social pediatric centers should be established, which are open for all chronic complex RND and not only for people with intellectual or multiple disabilities
Exchange and cooperation between rare disease centers and other partners in the health care sector (Statements 31–36)	Cooperation and mutual exchange between ERNs, the DRN, DASNE and non-ERN hospitals as well as patient organizations, e.g. with respect to patient registries Raising awareness for DRN-RND/ DASNE to non-specialist centers / private practices through established periodicals, web sites and educational events Development by the DRN-RND/ DASNE of clear and easy-to-use pathways to access centers for RND for physicians Training courses / CME for non-expert treating physicians, e.g. on the initiation of human genetic diagnostics
Databases (Statements 37–38)	Documentation of RND in the hospital information system in outpatient and inpatient settings at all care facilities using Orpha codes Uniform data collection of disease-identifying data (e.g. Orpha codes), health status data and disease progression data across all hospital clinical information systems Uniform deep data collection in the centers for RND for the identification of specific groups of RND to facilitate personalized treatment and research

(*CME* Continuous medical education, *DASNE* Deutsche Akademie für Seltene Neurologische Erkrankungen (German Academy for rare neurological diseases), *ERN* European reference network; *DRN* Deutsches Referenznetzwerk (German reference network), *RND* Rare neurological diseases, *SOP* Standard operating procedure).

## Discussion

To the best of our knowledge, we have developed the first recommendations for optimal interdisciplinary management and healthcare settings for patients with rare neurological diseases on the basis of an adapted Delphi procedure involving a large interprofessional expert group. The recommendations can be taken as a guidance as to how and in which setting care for patients with RND should be provided. All recommendations were fully consented by our interprofessional expert group, which also included patient representatives. This level of agreement suggests that our recommendations provide important guidance for the development and delivery of high-quality RND care and we strongly recommend their use in realizing and planning the RND care provision.

Our consensus recommendations offer broader generalizability to all rare diseases. As far as we are aware of, no comparable study has been performed as yet for any rare disease. The recommendations highlight that the focus of RND care is on interprofessional and interdisciplinary, patient-centered and expertise based informed care delivery. Common themes that were mentioned across more than one main topic and which could thus be deemed as the very essence for RND care provision were the following:

- Interdisciplinary and interprofessional care provision
- Continued medical education for RND experts and non-experts
- Importance of neurologic and neurogenetic expertise and expertise based decisions
- Empowered participation and contribution of patients and patient advocacy organizations
- Networking/cooperation between different players in the field of RND
- Digital infrastructure including digital patient records, which are accessible to the entire interdisciplinary and interprofessional team involved in the care of a RND patient as well as use of Orpha codes for coding of RND patients,
- Development of standard operating procedures for all RND related activities
- Adequate funding of care services and structures, and
- Structured and validated public information on RND expertise centers.

This study has several strengths. First, the resulting recommendations owe their credibility to the use of a modified Delphi procedure [6]. The authors have set clear standards for the conducting and reporting of the Delphi study, including the appointment of independent researchers to coordinate the study, the presence of a clear consensus criterion, clear descriptions of how the synthesis of responses in one survey round was used to design the subsequent round, and the review and approval of the final draft by an external board before publication and dissemination.

Second, the Delphi method allowed the involvement of a network of 14 interprofessional experts. These participants had various professional backgrounds and work settings. In the expert group, we also included six patient representatives. Our response rate of more than 80% indicates that the risk of selection bias is low. Third, the high degree of consensus in the expert groups regarding topic selection as well as derived recommendations contributes to the validity of our findings.

We acknowledge the following limitations of our study. For the study we performed we could not find an appropriate literature basis. Secondly, as healthcare for RND patients in Germany is currently changing, our recommendations might need to be updated considering the effects of these changes. Finally, recommendations need validation in the actual healthcare setting. Whether the use of the recommendations will, in fact, improve care provision is a matter that warrants further study.

As future steps, we recommend the dissemination, and implementation of these recommendations for use in practice and policy making. We also suggest evaluating the use of these recommendations in clinical practice, and their usefulness to change the healthcare system.

## Conclusions

Our large interprofessional expert group came to a consensus on recommendations for RND. These recommendations represent an important first step in providing instructions and orientation with a view to the care that should be provided for RND patients. We hope these recommendations will have a catalytic effect to benefit patients and their relatives by changing the provision of care in the German healthcare system, thus contributing to improved quality of life for RND patients and other patients with rare diseases in Germany. Future implementation of these recommendation in care practice depends to a large extent on the systematic integration of specific care pathways and expertise networks such as the ERN-RND in the healthcare system as well as on respective resource allocation.

## Methods

### Determination of the most important topics

In May 2020, after studying the literature and finding no review or publication addressing the question of optimal interdisciplinary management and healthcare settings for patients with rare neurological diseases (RND), as a first step an interdisciplinary group of 34 experts including patient representatives was contacted and asked to propose topics that are important for optimal care of patients with rare neurological diseases.

Potential panel experts (including patient representatives) were identified through their involvement in DASNE or through the professional networks of the members of the taskforce. In the selection process, we aimed for an interdisciplinary group of RND experts. The invited panelists were experts in RND research, practice, and policy, with backgrounds in medicine, social counseling, physiotherapy, ergotherapy, speech therapy and policy. Invited panelists also included five patient representatives from German patient advocacy organizations like the German Heredo-Ataxia Society.

In the letter that was used to contact the experts we explained the goal and the process of the study and asked an open question for topics that are important for the care of RND patients. The online questionnaire was answered by 17 experts that formed the expert panel for the ranking of the topics (see below). Subsequently, we structured these replies into main topics and subtopics. This process yielded 11 main topics each containing a number of subtopics.

### Delphi round 1 and 2—ranking of topics

In June and July 2020, in the first Delphi round the determined main topics and subtopics were sent to the same expert panel through an online questionnaire. For the ranking of the main topics and subtopics, panelists were asked to rank the perceived importance of both main topics and related subtopics on a 6-point Likert scale (1 = least important to 6 = most important). The panelists' responses were used to calculate the levels of importance. Importance was indicated by a median score, which represents the 50th percentile value of opinions.

In August and September 2020, panelists received the median score of all topics together with the score they had given in the first Delphi round and were requested to re-assess their respective scoring. To maintain conformity between rounds, only those panelists who responded to the online questionnaire in the first Delphi round (n = 14) were asked to respond to the ranked topics in the second Delphi round. Again, panelists could indicate the perceived importance of both main topics and related subtopics on the same 6-point scale. All 14 panelists who responded in the first Delphi round, also responded in Delphi round 2. As we observed no major rating differences, especially with regard to downgrading but rather a ceiling effect, from round 1 to round 2, we decided to terminate the Delphi process after round 2.

Main topics were included in the recommendation development after round 2 if median ratings were ≥ 5 or if a subtopic received a median rating ≥ 5. If a main topic did not receive a median rating ≥ 5 but a linked subtopic or different subtopics did, the recommendation development focused on the respective subtopic(s).

### Formulation of recommendations on most important topics

The prioritized main topics were used to form small interdisciplinary working groups composed of three to five members of the expert group that were tasked to formulate draft recommendations relating to the identified topics. Working groups were formed on the following main topics: Structure of the care facility, ensuring neurological core expertise and core mission, composition of the interdisciplinary team, diagnostics, case conferences, continuous care and therapy development, translation, patient advocacy organizations, health policy, exchange and cooperation between rare disease centers and other partners in the health care sector and databases. We provided an example recommendation to inform the recommendation drafting and discussion in the working groups. To the working groups, we invited the initial larger expert group that we contacted in round 1. 37 experts contributed to the working groups and their respective drafting of recommendations and to the consensus process. All draft recommendations were received by May 2021 (Additional file 1).

### Consensus on recommendations

In June and July 2021, we organized a consensus process consisting of three steps. All draft recommendations were presented, thoroughly discussed and adapted in two online consensus meetings, in which the entire expert panel participated.

After the two meetings we shared the current stage recommendations with the panel and accepted further comments for two weeks. The set of recommendations was then circulated and approved by the entire panel.

### Supplementary Information

**Additional file 1.** Full formulation of recommendations for optimal interdisciplinary management and healthcare settings for patients with rare neurological diseases.

## Data Availability

The data that support the findings of this study are available in the supplementary material at the end of the manuscript.

## Appendix 1: Results of the Delphi process used for the ranking of the topics relevant for RND care provision

**Table:**

Main topics	Median vote Delphi round 1	Median vote Delphi round 2
Structure of the care facility	5	5
Ensuring neurological core expertise and core mission	6	6
Composition of the interdisciplinary team	5	5
Diagnostics	4	6
Continuous care and therapy development	4,5	5
Case conferences	4	4
Exchange and cooperation between rare disease centers and other partners in the health care sector	4,5	5
Patient advocacy groups	4	5
Databases	5	4,5
Translation	4,5	5
Health policy	4	4,5

**Table:**

Subtopics	Median vote Delphi round 1	Median vote Delphi round 2
*Structure of the care facility*		
Requirements planning	4,5	4
Quality criteria/certification	4	4
Remuneration/time	5	5
Spatial equipment incl. therapy rooms	5	5
Therapeutic supplies	4	4
Integration into the health care system/establishment of cross-sector care pathways	5	5
*Ensuring neurological core expertise and core mission*		
Specialized training	6	6
Continuing education and training	5	5
Promotion of young talent	6	6
Expertise for defined rare diseases/disease groups	6	6
*Composition of the interdisciplinary team*		
Neurology, Neuropediatrics, Cognitive Neurology, Neurogenetics, Neuropathology	6	6
Neuroradiology/Nuclear Medicine	5	5
Other medical specialties, e.g. cardiology, orthopedics	4	4
Speech therapy, occupational therapy, physiotherapy	5	5
Nursing care	5	5
(Neuro)Psychology	5	5
Psychosocial/social-medical counseling	5	5
Genetic counseling	5	5
Medical assistant	4	4
*Diagnostics*		
Special functional diagnostics, e.g. physiotherapy	4	4
Next Generation Sequencing (reimbursement, evaluation)	6	6
Prenatal/preimplantation diagnostics	4	4
*Continuous care and therapy development*		
Interdisciplinary planning	5	5
Clinical trials	5,5	5,5
Standardized scales and scores	5	5
Quality of life	5	5
*Case conferences*		
On site (structure and remuneration)	5	5
Digital (remote) case conferences (structure and remuneration)	4	4
*Exchange/cooperation between ZSEs and other partners in the health care sector*		
Exchange and cooperation between expertise centers for rare diseases	5,5	5,5
Between expert centres for rare diseasese and Psychiatry/Pain medicine	4	4
Between expert centres for rare diseases and medical centres for adults with multiple disabilities (joint consultation hours, remuneration)	4,5	4,5
Cross-sectoral exchange and cooperation	4,5	5
*Databases*		
Intra-rare disease centers	4	4
Inter-rare disease centers	5	5
Disease registry incl. biobanking, trial-readiness	6	6
Health insurance companies, making data available for research purposes	4	4
*Health policy*		
Social discourse on diagnostics and treatment costs	5	5
Political lobbying	5	5
Gene Therapy	5	5
International symbol for people with movement disorders (analogous to the sign for the blind) to prevent discrimination and stigmatization	4	4
