# Supplementary material for: Recommendations for optimal interdisciplinary management and healthcare settings for patients with rare neurological diseases
Source: Orphanet J Rare Dis. 2024 Feb 13;19:62. doi: 10.1186/s13023-024-03023-1 (PMC10863275; doi:10.1186/s13023-024-03023-1)
Supplement: Supplementary file 1 — Additional file 1. Full formulation of recommendations for optimal interdisciplinary management and healthcare settings for patients with rare neurological diseases. [file 13023_2024_3023_MOESM1_ESM.docx]

**Supplement - Full formulation of recommendations**

**Structure of the care facility**

*Statement 1:* For the care of people with rare neurological diseases, sufficient and sustainable resources (space and funding) must be provided within the hospital organization, especially at university hospitals. Medical structures and service offerings can vary and can range from the specialist out- patient clinics dedicated to specific questions related to a particular disease, its diagnostics or special therapy options, to the interdisciplinary center, which provides comprehensive outpatient care for patients with rare and complex conditions. Specialized centers with an expertise in rare neurological diseases should fulfill the NAMSE criteria for specialized centers (<https://www.namse.de/fileadmin/user_upload/logos/Anforderungskatalog_an_Typ_B_Zentren_120419.pdf>) and meet the minimum number criteria of the European Reference Networks.

*Statement 2:* The care of people with rare diseases is usually provided in a modular way in current hospital structures. This means that patients are assessed by one discipline at a time. Non-medical therapy is usually only prescribed. Interdisciplinary care may not occur, or may occur only with difficulty. This care may work in procedurally clear diseases, but it does not do justice to patients with rare diseases with often complex requirements and necessary coordination needs. For example, an adolescent with generalised dystonia who requires coordination between paediatrics, neurology, neurosurgery, orthopaedics, rehabilitation technology and occupational and physiotherapy. In order to coordinate the necessary therapeutic steps between the different disciplines to achieve the therapy goals formulated together with the patient, joint consultation hours are the most sensible and efficient measure for the patient. Statement interprofessional consultations must be coordinated by the centres for rare diseases.

*Statement 3:* Currently, neurological training in university and non-university institutions is primarily characterized by the diagnosis and management of revenue-relevant common diseases, in particular stroke, classic Parkinson's syndromes, and common immunological diseases, especially multiple sclerosis. The main focus is on inpatient care and billing according to the DRG system. Inpatient care of rare neurological diseases as well as specialized outpatient clinics for defined mostly rare neurological diseases have been neglected. Thus, large gaps have emerged in the training and systematic study of the numerous rare neurological diseases, making rational diagnosis and management of rare neurological diseases difficult. A curriculum in which rotations in special outpatient clinics for rare neurological diseases and participation in structured case conferences are an integral part should be strived for.

**Ensuring neurological core expertise and core mission**

*Statement 4:* The management and coordination of a center for rare neurological disorders should be the responsibility of a neurological specialist with several years of clinical expertise in rare neurological disorders.

*Statement 5:* Neurogenetic expertise should be available in the center. For this purpose, the head of the center or another experienced member should have acquired the additional qualification "specialized human genetic counseling" or there should be a structurally and personnel sustainable cooperation with a human genetics department.

*Statement 6:* Whenever possible, medical students and/or residents in training should be actively involved in the clinical care of patients with rare movement disorders to ensure early quality training.

**Composition of the interdisciplinary team**

*Statement 7:* The composition of the interprofessional team requires the following professional groups: Medicine (neurology, neuropaediatrics, cognitive neurology, neurogenetics/genetic counselling, neuropathology, neuroradiology/nuclear medicine), health care professions (speech therapy, occupational therapy, physiotherapy, (neuro)psychology), as well as health care and nursing and psychosocial/social medical counselling.

The involvement of patients' relatives and representatives of self-help is indispensable.

*Statement 8:* The care of persons with rare diseases requires an interprofessional person-centred orientation of the entire care process. In the context of Shared Decision Making, this means that patients, as experts for their own life situations and disease concerns, are the starting and reference point for all decisions. Instruments such as Goal Attainment Scaling should be used to involve patients in the goal-setting and evaluation of the care process. Interprofessional approaches should support the empowerment of patients and enable them to deal with their own health-related problems and to advocate for their interests in the social context.

*Statement 9:* Coordinated and cross-sectoral care requires networking of the different health care facilities and a coordinating body. To this end, structures and binding procedures must be created that guarantee continuous interprofessional cooperation in line with needs. The centres for rare diseases assume a central coordinating position for networking with other decentralised outpatient care (e.g. general practitioners, outpatient therapy practices).

The basis for interprofessional cooperation is a standardised interprofessionally coordinated documentation system as well as organisational processes with regularly anchored case conferences.

A common digital infrastructure that facilitates the exchange of care and patient data would be desirable.

*Statement 10:* Demand-oriented health care for people with rare diseases requires interprofessional training of the health care professions from the very beginning, as well as the establishment of regular interprofessional continuing education. A continuous cross-centre exchange on diagnosis and treatment processes must be established in everyday care. Appropriate structures must be provided for this, with continuous cross-centre interprofessional networks with mono- and interprofessional working groups and expert circles. Collegial counselling and case supervision should be used for special challenges in the care process.

*Statement 11:* In order to support people with rare diseases in their participation in the areas of life in the best possible way, interprofessional cooperation should not only be limited to actors in the healthcare system, but should also be aimed at municipal structures in cities and communities. This includes, for example, support in finding and/or adapting housing or integration into the labour market through job coaching.

**Diagnostics**

*Statement 12:* In accordance with the National Action Plan for Rare Diseases (NAMSE) case conferences are considered a crucial and integral activity of all Centers for Rare Diseases. Statement should be installed at an early diagnostic stage.

They should be planned, organized and documented according to well-defined internal standard operating procedures, also taking into consideration the regulations of the Gemeinsamen Bundesausschuss on Centers for Rare Diseases (§136c, para 5 of the SGB V).

If a rare neurological disease is suspected, an interdisciplinary case conference should be convened early in the diagnostic workup. This body must be interdisciplinary and interprofessional (including e.g. occupational therapy, physiotherapy), with both diagnostic and clinical expertise in the required disciplines. The clinically supervising discipline presents the patient case. Together with the individually relevant disciplines, consultations are held and decisions are made on the further diagnostic and therapeutic procedures. The indication to complete a missing or incomplete pre-diagnosis should be given generously. If the disease remains unclear, contact should be made at an early stage with supraregional expert panels.

*Statement 13:* The aim of finding the cause of a rare disease is first and foremost a targeted therapy as early as possible, to significantly improve the symptoms. In addition, a statement about the prognosis and, the course up to and life expectancy is desirable. For some genetic diseases, specific screening examinations can be offered, e.g. if, in addition to the manifest symptoms, an involvement of other organ systems is to be expected, or if the development of benign or malignant tumours is to be expected. In Statement cases, preventive examinations are of particular importance.

In addition, in a number of genetic diseases, certain factors that lead to a worsening of the symptoms can be identified and subsequently avoided through correct diagnosis assignment.

Furthermore, the importance of clarifying the causes for the process of psychological processing and acceptance of the disease should not be underestimated. Not infrequently, this also serves to reduce feelings of guilt - especially in the case of parents of children with the disease.

In rare (neurological) diseases, genetic diagnostics often play an essential role. The standard diagnosis for suspected genetic diseases should be an exome analysis (analysis of the coding gene regions using NGS), which should be extended by further genetic analyses (e.g. methods for analysing unstable repetitive DNA sequences) if necessary and in accordance with the suspected diagnosis. The recommendation for an exome analysis and its evaluation is made either by interdisciplinary case conferences with the participation of human genetics or by a specialist in human genetics.

Not infrequently, neuroradiological diagnostics also point the way. In interdisciplinary case conferences, the existing neuroradiological diagnostics should be reviewed and interpreted by a neuroradiologist experienced in the field. In the case of incomplete or insufficient pre-diagnostics, a generous effort should be made to complete the imaging, specifying the necessary modality and recommendations for performing the examination in an appropriately experienced institute.

Based on the decision of the expert panel (see thesis 1), other disciplines or professional groups are consulted. Statement include both other disciplines of human medicine (e.g. psychiatry) and other health professions (e.g. speech therapy, psychology).

**Case conferences**

*Statement 14:* Most patients with rare neurological diseases require interdisciplinary care due to multisystemic affections. This need must be met by interdisciplinary consultations and case conferences. They should be planned, organized and documented according to clearly defined internal standard operating procedures (SOPs), also considering the regulations of the Gemeinsamen Bundesausschusses zu den Zentren für Seltene Erkrankungen (§136c, para. 5 SGB V). The SOPs should be harmonized across centers in the German Reference Network for Rare Neurological Diseases in collaboration with the German Academy for Rare Neurological Diseases (DASNE). As a minimum requirement, case conferences should include three different specialties. The participation of a neurologist is mandatory. Additional disciplines will be determined based on the cases discussed. Case conferences should be based on structured case presentations including and presenting all relevant medical information and documented in the local hospital information / management system. Case conferences can be conducted as on-site or teleconferences. For this purpose, easy-to-use, nationally/European standardized and data-protection-secure systems for the exchange of patient data (including findings and videos) and for video conferences must be made available.

*Statement 15:* Due to their complexity, interdisciplinary case conferences require a new remuneration system. This should consider the time required and the number of disciplines involved. Corresponding documentation must be kept as proof.

*Statement 16:* Experts in rare diseases are scarce in all disciplines and are difficult to recruit, especially for external patients, due to their workload. CSEs should maintain lists of experts who are willing to participate in external case conferences. Since this will only be possible outside working hours, appropriate, personal compensation should be provided

**Continuous care and therapy development**

*Statement 17:* Finding specialists for specific rare diseases by patients or relatives on the basis of the subjective perception of key symptoms by patients / relatives can be improved through structured Internet offers. Corresponding internet offers would have to be developed. Analyzing patient search queries could be helpful in developing such tools.

*Statement 18:* The early consultation / use of experts in the assessment of patients with diagnostically unclear rare diseases by practicing neurologists or general practitioners / family physicians should be improved / facilitated. This could be realized through remunerated participation in (online) case conferences. In addition to monetary incentives, participation in case conferences should be upgraded by continuing education points or similar. The aims are the realization of rational / resource-saving diagnostics and the shortening of the time to diagnosis.

*Statement 19:* Reducing budgetary restrictions (risk of regression) for general practitioners for patients with rare diseases (e.g. through a positive list of drugs / prescriptions by national expert committee) can significantly improve the care situation for patients with rare diseases and the burden on relatives.

*Statement 20:* Care networks (speech therapists, occupational therapists, physiotherapists, neurologists, psychiatrists) for specific rare diseases can significantly improve the care situation for patients with rare diseases and the stress on relatives. The development of curricula and further training catalogs for specific rare diseases can facilitate the establishment of care networks.

*Statement 21:* National registries / cohort studies for patients with rare diseases as trial-ready cohorts can facilitate the conduct of clinical studies and the development of new standards of care.

*Statement 22:* National platforms for communication and standardization of individual healing attempts can facilitate the translational development of new therapy concepts while generating hypotheses.

**Translation**

Statement 23: Increasing knowledge of the genetic basis and pathomechanisms of rare neurological diseases is bringing the development of targeted therapies closer. This makes safe and early diagnostics all the more important. Standards and recommendations for diagnostics must therefore be developed. This concerns genetic diagnostics, possibly also neonatal screening; it also concerns the identification of biochemical biomarkers as well as neuroimaging parameters.

Statement 24: Increasing knowledge of the genetic basis and pathomechanisms of rare neurological diseases allows the identification of molecular targets and the development of targeted therapies. Translation from animal models to clinical development and application is important to promote. The sometimes very small numbers of cases in the individual disease entities, together with the often highly variable course of the disease, make the development of clinical studies challenging. It is therefore all the more important to develop standards and recommendations for clinical description as well as for clinically meaningful and appropriate outcome parameters.

**Patient advocacy organizations**

*Statement 25:* Information is exchanged, therapy decisions are made jointly and on an equal footing. Decisions are discussed and jointly supported by the attending/treating doctors, therapists as well as those affected and their families or carers. Those involved explain and discuss aspects and concerns openly and explain their preferences. Decisions are made jointly with mutual responsibility and acceptance.

Statement 26: The charter of EACH, the European Association for Children in Hospital, should be heeded. https://each-for-sick-children.org/each-charter/

*Statement 27:* Continuous care by experienced doctors with regular specialised consultations (if possible once a week or more frequently) and the possibility of emergency treatment and inpatient admissions including intensive medical care must be guaranteed within the centres.

**Health policy**

Statement 28: Political and administrative decisions concerning the care of patients with rare neurological diseases should be legitimised by referring to the existing expertise and the available knowledge on rare neurological diseases. It should be the declared goal of the care experts to make the available knowledge communicatively available for political and administrative decision makers with the involvement of the patient organizations and thus to increase the "accuracy" of the decisions.

*Statement 29:* Patients with SNE should be cared for across sectors. The predominant separation of medical care into outpatient and inpatient care, which has been established in Germany for many decades, often leads to both an excess of diagnostics and delays in diagnosis on the one hand, and often to uncoordinated management after diagnosis on the other hand, due to insufficient exchange and communication between the service providers. To set the course for diagnostic and therapeutic decisions, cross-sectoral structures should be established to counteract this. Statement include, in particular, case conferences and cross-sectoral patient files, which should be open to both staff of the CSEs and colleagues in the outpatient sector. Appropriately secure case conferences and patient record portals should be used for this purpose. Appropriate flat rates should be granted, especially for the case conferences, in order to create incentives for participation, which in turn forms the basis for specialist certificates.

Patient files should be kept by the CSEs with the involvement of the patients, who should exchange information with each other if necessary. The aim is to consolidate previous diagnostics, to avoid unnecessary further diagnostics and to draw up an overarching treatment plan.

Statement 30: Children, adolescents and adults with rare neurological disorders (RND) are particularly dependent on optimal care according to the bio-psycho-social model. This requires interprofessional interdisciplinary care, such as that established in social paediatric centres (SPC). For the optimal care of paediatric patients with RNDs, Centres for Rare Neurological Diseases in Germany should therefore work closely with at least one of the 160 SPCs. For adults with RND, similar comprehensive care in Medical Treatment Centres for Adults (MTCA) should be demanded. The MTCA should be opened for all chronic complex RND, not only for people with intellectual or multiple disabilities. Centres for Rare Neurological Diseases should then work closely interprofessionally with at least one of the MTCA. Accompanying persons / carers should be included in the care.

**Exchange/cooperation between Centre for Rare Diseases and other partners in the health sector**

Statement 31: European Reference Networks (ERNs) have developed into an important institution for improving patient care in the field of rare diseases, which is now having more and more national impact. For example, the ERNs are mentioned in the G-BA Centre Regulations, where networking is emphasised (Supporting Reasons for Centre Regulations, Annex 1 - ZSE §1 (1) 3.). Networking with the ERN-RND is best institutionalised via the German ERN members in the corresponding German Reference Network (Deutsches Referenznetzwerk; DRN) or DASNE, whereby university and other hospitals not participating in the ERN but relevant to the area as well as patient organisations should be part of it. Information should be provided regularly and cover all relevant ERN areas. It is important to ensure an information flow in both directions.

Statement 32: Close cooperation between the DRN-RND/DASNE and ERN-RND can improve patient care in the field of rare neurological diseases on a large scale. National projects should be tested for European application and opened up where possible, while at the same time the progress made in the ERN should be transferred to national application where appropriate and necessary. This includes, for example, the use of the CPMS, taking into account the Statements developed for case conferences - where possible - or the use of Europe-wide registries.

*Statement 33:* Family physicians and specialists in private practice/therapists /practice staff/specialists from smaller hospitals are usually the first to see patients with suspected rare disease and thus have a decisive influence on the length of the diagnostic pathway. Raising awareness is therefore crucial. To achieve this, all channels should be used to make the DRN-RND/ DASNE better known (e.g. Deutsches Ärzteblatt and other publications relevant to this professional group and the development of a web presence) and the ways to contact the DRN-RND/ DASNE (see statement 4).

Statement 34: The DRN-RND/ DASNE should develop clear pathways for who can contact whom and how. The target is to allow only physician and no direct patient inquiries and to develop a short form with the central questions (model could be the form used in the Translate NAMSE/ZSE-DUO project and developed on the basis of the GP association). This should then be publicized through the channels mentioned in Thesis 3.

Statement 35: Educational events, e.g., in DASNE, FAKSE, or by others that directly address practicing colleagues, may be an adequate means of raising awareness. This could include case presentations based on the "from common complaints to rare disease" format and should be linked to the possibility of receiving continuing education credits.

Statement 36: Since, according to the G-BA center regulations, ZSEs must have access to human genetic expertise - ideally as an integral component - and patients generally make contact via a physician, it is considered expedient to also focus networking activities on the practising sector (see corresponding Statements). In particular, training courses on the initiation of human genetic diagnostics should be offered to physicians in private practice.

**Databases**

*Statement 37:* For the recording and documentation of rare neurological diseases in the hospital information system, diagnostic coding of all patients with the Orphacode at the disease entity level is necessary. This coding should be done in outpatient and inpatient settings at all care facilities. This not only enables the visibility of rare neurological diseases, but also facilitates the investigation of and decision-making on questions relevant to care, as well as the transition to specific research registries.

In this context, diagnostic coding must not be compromised with the purpose of billing, as is currently the case for coding with ICD10 GM.

*Statement 38:* The inhomogeneity of the various clinical information systems and the very limited networking represent a major challenge in the care and clinical research of rare diseases. Therefore, firstly, uniform data collection of disease-identifying data (coding of rare diseases (e.g. Orpha code)), health status data and disease progression data across all hospital clinical information systems (SAP, ORBIS, etc.) is required. Secondly, uniform deep data collection in the centres for rare diseases is necessary so that specific groups of patients with rare diseases can be identified across sites via medical informatics networking in order to enable personalised treatment and research approaches.
